# Factors influencing the level of performance of patient safety nursing activities among hospital nurses

**DOI:** 10.7586/jkbns.24.010

**Published:** 2024-05-28

**Authors:** Hyun-Ju Beak, Gisoo Shin

**Affiliations:** 1 Chung-Ang University, Gwangmyeong hospital, Gwangmyeong, Korea; 2 College of Nursing, Chung-Ang University, Seoul, Korea

**Keywords:** Patient safety, Nurses performance evaluation, Hospitals, Nurses

## Abstract

**Purpose:**

This study investigated the performance of patient safety activities among hospital nurses and aimed to identify the factors influencing their performance of these activities.

**Methods:**

It employed a descriptive survey design, targeting 131 nurses currently working in hospitals. The data collection involved posting a guide to the study on an online social network for nurses (NURSECAPE) and recruiting nurses who understood the content and agreed to participate in the survey. The survey was conducted through a self-reporting method via a URL provided to research participants, and the data collection period was from August 11 to September 11, 2019.

**Results:**

The results revealed that 46.6% of the participants had experienced patient safety incidents, with falls being the most common. The factors influencing the performance of patient safety nursing activities among the participants were found to be the type of medical institution, community orientation, and environmental suitability in organizational health. These factors explained 38.5% of the variance.

**Conclusion:**

Based on these findings, it appears crucial to explore strategies for improving organizational health tailored to the characteristics of each hospital to facilitate better performance of patient safety activities among hospital nurses. Furthermore, subsequent studies are needed to objectively evaluate the adequacy of patient safety activity performance according to the size of the hospital.

## INTRODUCTION

Patient safety refers to the absence of accidental or preventable harm during the process of medical care delivery. Hospitals, being complex organizations where individuals of various professions concurrently perform tasks under time constraints and prolonged focus, are susceptible to patient safety incidents [1]. The concept of patient safety emerged in 1999 with the publication of the Institute of Medicine's report "To err is human: building a safer health system." According to the report, it is estimated that up to 98,000 people die each year due to medical errors in hospitals, a number higher than deaths from car accidents, breast cancer, and AIDS. It is speculated that the number of patients dying each year due to medication errors within hospitals may be even higher than the reported figures [2]. Moreover, while the probability of death from an accident during air travel is 3 million to 1, the likelihood of patient death due to preventable medical errors is 300 to 1, making healthcare, perceived as riskier than aviation or nuclear industries, significantly more hazardous [3]. As awareness of these issues grew, patient safety gained societal attention and was recognized as a critical issue in healthcare [4]. Ensuring that patients receive safe treatment in a secure environment is one of their fundamental rights. As various safety incidents result in decreased quality of care and financial losses for healthcare institutions, the importance of patient safety continues to rise [5].

Although patient safety is the responsibility of all hospital staff, nurses, who establish close relationships with patients and provide care around the clock, bear significant responsibility within the hospital's safety management domain [6]. Patient safety nursing activities involve establishing clear goals to enhance patient trust and safety during the care process. These activities include developing preventive measures and evaluating their effectiveness to prevent accidents and improve patient safety [1-4]. Reporting patient safety incidents is the initial step in these activities. Through reporting, recurring issues in medical settings are identified, addressed, and managed promptly through experiential learning and action [7]. In South Korea, since the enactment of the Patient Safety Act in 2016, healthcare institutions are required to voluntarily report patient safety incidents to the Korea Patient Safety Reporting & Learning System (KOPS). The number of monthly reports has been increasing steadily, leading to the establishment of a national system for analyzing patient safety incidents and preventing their recurrence [8].

Patient safety incidents are not solely individual responsibilities but often reflect systemic organizational issues. Preventing such incidents requires an open organizational culture. However, in clinical settings, nurses, who are closest to patient care, may be scapegoated for incidents, leading to underreporting of patient safety incidents [9]. Furthermore, organizational health is crucial for adaptation, growth, and sustainability within the organizational environment. Modern organizations, including hospitals, require careful management to adapt to diverse purposes, specialization demands, and rapid changes in order to survive [10]. Organizational health is characterized by maintaining member autonomy, fostering effective communication to boost morale, maximizing organizational effectiveness through supportive structures and systems, and promoting teamwork among nurses, enhancing job satisfaction and enabling them to provide higher levels of care [11]. Research suggests that nurses who perceive organizational health positively tend to be more engaged in the organization and experience greater job satisfaction [12]. Additionally, organizational commitment and job satisfaction have been found to positively influence patient safety nursing activities [13]. Although various studies have confirmed the positive impact of organizational health on patient safety nursing activities, generalization remains limited [10].

Therefore, this study aimed to identify the types of patient safety incidents experienced by nurses working in hospitals, assess their performance in patient safety incident activities, and determine the factors influencing patient safety incident activities. The specific objectives of this study are as follows: First, to identify the patient safety incident experiences perceived by the subjects of this study, organizational health, and the extent of patient safety nursing activities perceived by them. Second, to verify the differences in the extent of patient safety nursing activities according to the characteristics of the subjects. Third, to elucidate the influencing factors on the extent of patient safety nursing activities of the subjects.

## METHODS

### 1. Design

This study is a descriptive survey aimed at identifying the perception of patient safety incident experiences, organizational health, the extent of patient safety nursing activities, the relationship between these factors, and the influencing factors on patient safety nursing activities among hospital nurses.

### 2. Participants

The participants of this study were nurses currently employed in hospitals who directly provide nursing care to patients. The sample size was calculated using G*Power 3.1.9.4, a sample size calculation program based on Cohen's sample size formula. With a significance level of .05, power of .95, effect size of .15, and three predictor variables, the appropriate sample size was determined to be 119. Considering a dropout rate of 10%, a total of 133 nurses were surveyed.

### 3. Data collection

Data were collected from August 11th to September 11th, 2019. Nurses were informed about the study through the online nursing social network 'NURSCAPE (https:/www.nurscape.net)', and those who agreed to participate after understanding the content were targeted for the survey. The survey was conducted using the Google online survey URL link method, and it took an average of about 8 minutes to complete the survey. In this study, if participants withdrew during the research or if respondents were unfaithful, additional participants were recruited and surveys were conducted to maintain the sample size of 133 participants.

### 4. Measurements

#### Characteristics of research participants

The characteristics of the study participants include age, gender, marital status, education level, intention to change jobs, years of work experience, the size of the hospital they work in, the presence or absence of patient safety incident experiences in the past year, and the types of patient safety incidents experienced.

#### Organizational health

Organizational health refers to factors that influence patient safety activities, signifying the synchronization of members for this purpose [10]. Organizational health was assessed using a tool developed by Han and Jung [14], comprising 31 items categorized into environmental suitability (8 items), procedural suitability (9 items), vitality (8 items), and community orientation (6 items), rated on a 5-point Likert scale. Higher scores indicate a more positive perception of organizational health. The Cronbach's α reliability coefficient for this study was .97.

#### Performance level of patient safety nursing activities

The extent of patient safety activity performance refers to the adherence to guidelines for patient safety actions carried out within the hospital [4]. The tool developed by Han and Jung [14] based on the Certification Evaluation Criteria of the Korea Institute for Healthcare Accreditation was used. It consists of 32 items covering various aspects of patient safety nursing activities, rated on a 5-point Likert scale. The content of the tool consists of 4 items on accurate patient identification, 4 items on communication, 3 items on patient safety before surgery/procedure, 6 items on fall prevention activities, 5 items on hand hygiene and infection control, 2 items on fire safety and emergency management, 6 items on medication, and 2 items on facility and medical equipment management. Higher scores indicate a higher performance level of patient safety nursing activities. The Cronbach's α reliability coefficient for this study was .97.

### 5. Data analysis

The collected data were analyzed using SPSS statistics 23.0 (IBM Corp., Armonk, NY, USA).

Descriptive statistics including frequencies, percentages, means, and standard deviations were used to analyze the participants` general characteristics and research variables. Differences in variables according to the general characteristics of the participants were analyzed using t-tests and one-way ANOVA, with post-hoc tests conducted using the Scheffé test. Stepwise regression analysis was conducted to identify the factors that best explain the variation in patient safety nursing activities among hospital nurses.

### 6. Ethical considerations

Approval for this study was obtained from the Institutional Review Board of University (IRB No. 1041078-201906-HR-198-01 C) to ensure the protection of the research participants. The necessity, purpose, and process of the study were explained to all participants, and written consent was obtained voluntarily before participation. Participants were assured of the confidentiality of their responses and that the collected information would not be used for purposes other than the study. Participants were also informed that they could withdraw from the study at any time if desired. In addition, participants were provided with a nominal gift certificate for their participation.

## RESULTS

### 1. General characteristics and patient safety incident experiences

The total number of participants in this study was 133, with 122 females (91.7%) and 11 males (8.3%). The average age of the participants was 30.03 years, with 69 participants (51.9%) aged below 30 and 64 participants (48.1%) aged 30 or older. The majority of participant had a bachelor's degree (95 participants, 71.4%), and 82 participants (61.7%) expressed turn over intention at the time of the survey. The average total hospital work experience was 63.42 months, with an average recent unit working experience of 30.97 months. Among the participants, 62 (46.6%) had experienced patient safety incidents. The average number of patient safety incident experiences was 4.15 incidents, with falls being the most common (36 incidents, 50.0%), followed by medication errors (23 incidents, 31.9%) and injuries (3 incidents, 4.2%) (Table 1).

### 2. Level of patient safety nursing activities performance and organizational health

The average performance level of patient safety nursing activities among the study participants was 4.09 ± 0.63 points. Among these activities, hand hygiene and infection control had the highest average score (4.28 ± 0.74 points), followed by medication management (4.20 ± 0.73 points), preoperative/pre-procedural safety (4.16 ± 0.71 points), accurate patient identification (4.08 ± 0.73 points), fall prevention activities (4.06 ± 0.69 points), facility and medical equipment management (3.93 ± 0.80 points), and fire safety and emergency management (3.89 ± 0.75 points), with communication scoring the lowest (3.83 ± 0.77 points). Additionally, organizational health had an average score of 3.31 ± 0.66 points, with environmental suitability (3.40 ± 0.69 points), procedural suitability (3.32 ± 0.79 points), vitality (3.12 ± 0.75 points), and community orientation (3.10 ± 0.76 points) scoring sequentially lower (Table 2).

### 3. Differences in level of patient safety nursing activities performance according to general characteristics, patient safety incident experiences, and organizational health of participants

Significant differences in the performance level of patient safety nursing activities were observed according to turnover intention (t = 2.24, *p* = .027) and hospital classification (F = 6.44, *p* = .002) based on the general characteristics of the study participants. Additionally, statistically significant differences were found in organizational health and the performance level of patient safety nursing activities in terms of environmental suitability (t = 5.52, *p* < .001), procedural suitability (t = 5.74, *p* < .001), vitality (t = 4.89, *p* < .001), and community orientation (t = 5.86, *p* < .001). However, there was no statistically significant difference in the performance level of patient safety nursing activities according to the participants' experiences of patient safety incidents (Table 3).

### 4. Factors influencing the performance level of patient safety nursing activities

Before conducting stepwise multiple regression analysis to verify the factors influencing the performance level of patient safety nursing activities, a correlation analysis was conducted between organizational health and patient safety nursing activities. As a result, a positive correlation was found between organizational health and patient safety nursing activities(r = .53, *p* < .001). Based on this, turnover intention and hospital classification, which showed significance in the relationship between the general characteristics of the study participants and patient safety activities, were treated as dummy variables. Additionally, the four variables of environmental suitability, procedural suitability, vitality, and community orientation, which showed significant correlations with the organizational health, were included as independent variables, making a total of six variables for regression analysis. The regression model was significant (F = 21.65, *p* < .001). The Durbin-Watson statistic for autocorrelation was calculated to be 1.693, indicating no autocorrelation among the independent variables. Furthermore, the variance inflation factor for assessing multicollinearity among explanatory variables ranged from 1.32 to 1.59, indicating no multicollinearity issues among the variables. The stepwise multiple regression analysis revealed that the factors influencing patient safety nursing activities were the type of medical institution (general hospital) (β = 0.35, *p* < .001), community orientation of organizational health (β = 0.38, *p* < .001), and environmental suitability (β = 0.27, *p* < .001). These variables explained 38.5% of the variance in patient safety activities (Table 4).

## DISCUSSION

This study aimed to investigate the patient safety incident experiences, organizational health, and performance level of patient safety nursing activities perceived by hospital nurses, and to identify factors influencing patient safety nursing activities. Based on this, the results of the study are discussed.

In this study, it was found that a majority of the participants had experienced patient safety incidents, with falls being the most common type, consistent with the types of patient safety incidents reported by the Korean Institute for Healthcare Accreditation [15,16]. Therefore, guidelines and education for fall prevention considering the age and types of diseases of patients are necessary. Moreover, while immediate reporting of patient safety incidents according to the reporting system is crucial for patient safety, previous studies suggest that immediate reporting may have a negative impact on patient safety activities [17]. This might be because patient safety incidents often end up being attributed to individual nurse errors rather than being addressed as opportunities for improvement [18]. Thus, immediate feedback on patient safety incident reporting is essential, and instead of blaming or punishing nurses when incidents occur, there should be a focus on protecting nurses and strengthening. Furthermore, when patient safety issues arise, a fundamental root cause analysis is necessary, and systematic quality improvement activities and training are required to prevent patient safety incidents.

The organizational health reported by the study participants averaged 3.31 points on a scale of 5, which may be considered either high or low compared to previous studies [19]. The variation could be attributed to differences in the characteristics of the study participants and the organizational environments of the hospitals where they work. In this study, environmental suitability scored the highest in organizational health, while community orientation scored the lowest. This suggests that while participants are satisfied with their job duties, many still intend to change jobs [20]. Although the specific reasons for job intent were not investigated in this study, previous research suggests that factors such as shift work and complex interpersonal relationships are significant [21]. Since these factors are related to the lowest score in community orientation among the study participants, addressing the issues related to shift work and interpersonal relationships within the hospital environment should be prioritized to ensure continuous nursing activities.

The performance level of patient safety nursing activities among the study participants was high, with an average score exceeding 4 on a 5-point scale, higher than results from previous foreign studies [22]. This may be attributed to the increased awareness and importance of hospital patient safety among the public following various media reports on the group infant deaths at Ewha Womans University Mokdong Hospital in 2018 [23]. However, since the measurement of patient safety nursing activities in this study was self-reported, there is a possibility of response bias towards more positive responses. According to KOPS reports [7S], the number of patient safety incidents reported through self-reporting is much lower compared to the actual number of incidents. Therefore, the development of tools to objectively measure patient safety nursing activities and repeated studies using objective observation methods are required. Additionally, while hand hygiene and infection control activities were performed at the highest level among patient safety nursing activities in this study, communication scored the lowest. This discrepancy may be due to the emphasis on hand hygiene and infection control activities encouraged and monitored as part of domestic hospital accreditation evaluations [24]. On the other hand, communication is essential not only for doctor-nurse communication or nurse-patient communication but also as a prerequisite for patient safety nursing activities [25], necessitating exploration of various measures to activate communication within hospital organizations.

The factors influencing the performance level of patient safety nursing activities among the study participants were found to be the type of medical institution where they work and the subdomains of organizational health, specifically community orientation and environmental suitability, with an explanatory power of 38.5%. These results align with previous research indicating that working in a tertiary general hospital leads to higher performance in patient safety nursing activities [26], highlighting the significant influence of domestic hospital accreditation evaluations. Moreover, community orientation in organizational health, which signifies a collective consciousness among organization members, acts as a driving force for patient safety nursing activities [2]. Additionally, environmental suitability in organizational health indicates the ability to adapt well to internal and external environmental changes, which is crucial for assessing nursing competencies in performing safe patient care under unforeseen organizational circumstances, such as outbreaks of infectious diseases like coronavirus disease 2019 [27]. Therefore, creating a hospital environment that eliminates factors causing confusion in nursing duties and supports nurses in performing safe patient care amid various organizational changes is necessary.

The study participants were recruited through the nurse online social network, ‘NURSCAPE’, and self-reported surveys were conducted, which imposes limitations on generalizing the results. Nonetheless, despite these limitations, the significance of this study lies in confirming the influence of organizational community orientation and environmental suitability on nurses' performance of patient safety activities, contributing to evidence-based nursing practices.

## CONCLUSION

This study aimed to identify the factors influencing hospital nurses' experiences of patient safety incidents and their performance in patient safety nursing activities. The results of the study showed that the majority of the participants had experienced patient safety incidents, and the average score for the performance of patient safety nursing activities was high. Furthermore, the factors influencing their performance in patient safety nursing activities were found to be the size of the hospital where they currently work, community orientation in organizational health, and environmental suitability.

Based on the results of this study, it is imperative to explore ways to enhance organizational health by considering the unique characteristics of each hospital where nurses work. Additionally, further research is needed to objectively evaluate the performance of patient safety nursing activities based on the size of the hospital.

## Figures and Tables

**Table 1.** General Characteristics and Experiences of Patient Safety Incidents (N = 133)

Characteristics		n (%) or M ± SD	Range
Sex	Female	122 (91.7)	
	Male	11 (8.3)	
Age (yr)		30.03 ± 4.45	23-45
Marital status	Single	95 (71.4)	
	Married	38 (28.6)	
Education degree	Diploma	26 (19.5)	
	Bachelor	95 (71.4)	
	≥ Master	12 (9.1)	
Turnover intention	Yes	82 (61.7)	
	No	51 (38.3)	
Working experience (mon)		63.42 ± 45.49	2-240
Work duration in the most recent unit (mon)		30.97 ± 25.44	2-120
Position	Staff nurse	114 (85.7)	
	≥ Charge nurse	19 (14.3)	
Hospital classification	Tertiary	54 (40.6)	
	Secondary	45 (33.8)	
	Primary	34 (25.6)	
Experience of medical institution certification	Yes	64 (48.1)	
	No	69 (51.9)	
Experience of safety education in the past 1 year	Yes	98 (73.7)	
	No	35 (26.3)	
Experiences of patient safety incidents	Yes	62 (46.6)	
	No	71 (53.4)	
Number of patient safety incidents		4.15 ± 13.35	0-100
Type of patient safety incidents^†^	Fall	36 (50.0)	
	Medication error	23 (31.9)	
	Injury	3 (4.1)	
	Sore	2 (2.8)	
	Others	8 (6.0)	

M = Mean; SD = Standard deviation.

† duplicate response.

**Table 2.** Level of Performance of Patient Safety Nursing Activities and Organizational Health (N = 133)

Variables		M ± SD	Range
Performance of patient safety nursing activities	Hand hygiene and infection prevention	4.28 ± 0.74	2.20-5.00
	Medication	4.20 ± 0.73	1.83-5.00
	Operation/procedure prior patient safety	4.16 ± 0.71	2.33-5.00
	Accuracy of patient identification	4.08 ± 0.73	2.00-5.00
	Fall prevention	4.06 ± 0.69	2.00-5.00
	Medical equipment and facilities management	3.93 ± 0.80	1.00-5.00
	Fire safety and emergency management	3.89 ± 0.75	2.00-5.00
	Communication	3.83 ± 0.77	1.50-5.00
	Total	4.09 ± 0.63	2.25-5.00
Organizational health	Environmental suitability	3.40 ± 0.69	1.63-5.00
	Procedural suitability	3.32 ± 0.79	1.33-4.89
	Vitality	3.12 ± 0.75	1.25-4.63
	Community orientation	3.10 ± 0.76	1.50-5.00
	Total	3.31 ± 0.66	1.74-4.68

M = Mean; SD = Standard deviation.

**Table 3.** Differences in Levels of Performance of Patient Safety Nursing Activities according to General Characteristics, Patient Safety Incident Experiences, and Organizational Health (N = 133)

Variables			Performance	
			M ± SD	t/F (*p*)
General characteristics	Sex	Female	4.07 ± 0.65	0.77 (.444)
		Male	4.23 ± 0.44	
	Age (yr)	< 30.03	4.13 ± 0.62	0.77 (.441)
		≥ 30.03	4.04 ± 0.64	
	Marital status	Single	4.13 ± 0.60	1.16 (.249)
		Married	3.99 ± 0.70	
	Education degree	Diploma	3.97 ± 0.98	0.61 (.548)
		Bachelor	3.92 ± 0.78	
		≥ Master	4.17 ± 0.54	
	Turnover intention	Yes	3.99 ± 0.61	2.24 (.027)^†^
		No	4.24 ± 0.64	
	Working experience (mon)	< 63.42	4.09 ± 0.64	0.27 (.844)
		≥ 63.42	4.01 ± 0.74	
	Work durationin the most recent unit (mon)	< 30.97	4.08 ± 0.66	0.25 (.778)
		≥ 30.97	4.17 ± 0.47	
	Position	Staff nurse	4.10 ± 0.65	0.57 (.567)
		≥ Charge nurse	4.01 ± 0.54	
	Hospital classification	Tertiary^a^	4.27 ± 0.51	6.44 (.002)†
		Secondary^b^	4.09 ± 0.61	c < a
		Primary^c^	3.79 ± 0.72	
	Experience of medical institution certification	Yes	4.13 ± 0.52	0.75 (.452)
		No	4.05 ± 0.72	
Patient safety incident experiences	Experience of safety education in the past 1 year	Yes	4.15 ± 0.53	1.62 (.112)
		No	3.91 ± 0.83	
	Experiences of patient safety incidents	Yes	4.19 ± 0.84	1.33 (.272)
		No	4.16 ± 0.49	
	Number of patient safety incidents	< 4.15	4.10 ± 0.71	0.83 (.440)
		≥ 4.15	4.03 ± 0.80	
	Type of patient safety incidents	Fall	4.11 ± 0.79	0.35 (.771)
		Medication error	4.10 ± 0.85	
		Injury	4.07 ± 0.76	
		Sore	3.99 ± 0.45	
		Others	3.96 ± 0.53	
Organizational health	Environmental suitability	< 3.40	3.81 ± 0.68	5.52 (<.001)^†^
		≥ 3.40	4.36 ± 0.44	
	Procedural suitability	< 3.32	3.74 ± 0.71	5.74 (<.001)^†^
		≥ 3.32	4.35 ± 0.41	
	Vitality	< 3.12	3.82 ± 0.69	4.89 (<.001)^†^
		≥ 3.12	4.33 ± 0.46	
	Community orientation	< 3.10	3.70 ± 0.73	5.86 (<.001)^†^
		≥ 3.10	4.30 ± 0.45	

M = Mean; SD =Standard deviation.

^†^*p* < .05; Post hoc = ^a,b,c^Scheffe test.

**Table 4.** Factors Influencing the Performance of Patient Safety Nursing Activities

Variables	B	SE	β	t	*p*
(Constant)	1.91	0.25			
Hospital classification^†^					
Tertiary	0.45	0.11	0.35	4.11	< .001
Organizational health					
Community orientation	0.32	0.07	0.38	4.88	< .001
Environmental suitability	0.25	0.07	0.27	3.48	.001
F = 21.65, *p *< .001, R^2 ^ = .385					

SE = Standard error.

† dummy code (reference = primary).

## Data Availability

Please contact the corresponding author for data availability.
